# Comparison of Japanese and Scottish Mother–Infant Intersubjectivity: Resonance of Timing, Anticipation, and Empathy During Feeding

**DOI:** 10.3389/fpsyg.2021.724871

**Published:** 2021-10-14

**Authors:** Koichi Negayama, Jonathan T. Delafield-Butt, Keiko Momose, Konomi Ishijima, Noriko Kawahara

**Affiliations:** ^1^Faculty of Human Sciences, Waseda University, Saitama, Japan; ^2^Laboratory for Innovation in Autism and Faculty of Humanities and Social Sciences, University of Strathclyde, Glasgow, United Kingdom; ^3^Department of Child Studies, Shiraume Gakuen University, Tokyo, Japan; ^4^Faculty of Home Economics, Kyoritsu Women’s University, Tokyo, Japan

**Keywords:** intersubjectivity, interactional synchrony, Japan and Scotland, mother–infant relations, empathy in feeding, mouth opening

## Abstract

Feeding involves communication between mothers and infants and requires precise synchrony in a special triadic relationship with the food. It is deeply related to their intersubjectivity. This study compared the development of mother–infant intersubjectivity through interactional synchrony in feeding between 11 Japanese and 10 Scottish mother–infant dyads, observed at 6 and 9 months by video. Japanese mothers were more deliberate in feeding at an earlier age, whereas Scottish mothers were significantly more coercive than Japanese mothers at an earlier age. Japanese mothers brought the spoon to infants with a pause to adjust the timing of insertion to match their infants’ readiness, whereas this pause was not observed in Scottish mothers. Isomorphic mouth opening between mothers and infants was observed. This empathic maternal display is an important element of intersubjectivity in infant feeding that differed between Scottish and Japanese mothers. Scottish mothers’ mouth opening always followed their infants’ mouth opening, but about half of Japanese mothers preceded their infants. Further, the mouths of Scottish infants and mothers opened almost at the same time as spoon insertion. In contrast, Japanese mothers’ mouth opening did not co-occur with the insertion but was close to spoon arrival, a subtle but important difference that allows for greater infant autonomy. The time structure of Scottish mother-infant interactions was simpler and more predictable at 9 months than in Japan, where the structure was more variable, likely due to a stronger regulation by Scottish mothers. In conclusion, Scottish mother-infant intersubjectivity is characterized as more maternally reactive and mother-centered, whereas Japanese mother-infant intersubjectivity is characterized as more maternally empathetic and infant-centered. Cultural differences in intersubjectivity during feeding between Japan and Scotland are further discussed in relation to triadic relationships and parenting styles.

## Introduction

Feeding is essential for infant survival and health, and weaning is a biologically significant framework for understanding the development of infant independence from the mother (Trivers, 1974; Negayama, 1996). Human infants gradually become autonomous feeders over the first years of life in terms of hand and tool use (Connolly and Dalgleish, 1989; Norimatsu, 1993; Kawahara, 2005), and food choices (Pliner, 1994; Tovar et al., 2016; Cole et al., 2018).

Mothers assist their children’s feeding by providing food, cooking, and assisting with feeding when they are young and unable to manage it on their own (Wright, 1989). Such maternally supported infant feeding requires synchrony of intentions and actions on both sides, with attunement of these between mother and infant to ensure effective food intake, without choking. Interactional synchrony, or shared time, is fundamental to many domains of mother–infant intersubjectivity, and exists in the relationship even during the prenatal period – it is the basis of harmony between them (Feldman, 2003; Trevarthen et al., 2006). Infant feeding supported by the mother consists of a sequence of actions, or behavioral units, made with shared timing, and is one striking example of mother-infant intersubjectivity (Feldman, 2007).

### Empathy and Mother-Infant Intersubjectivity

**FIGURE 1:**
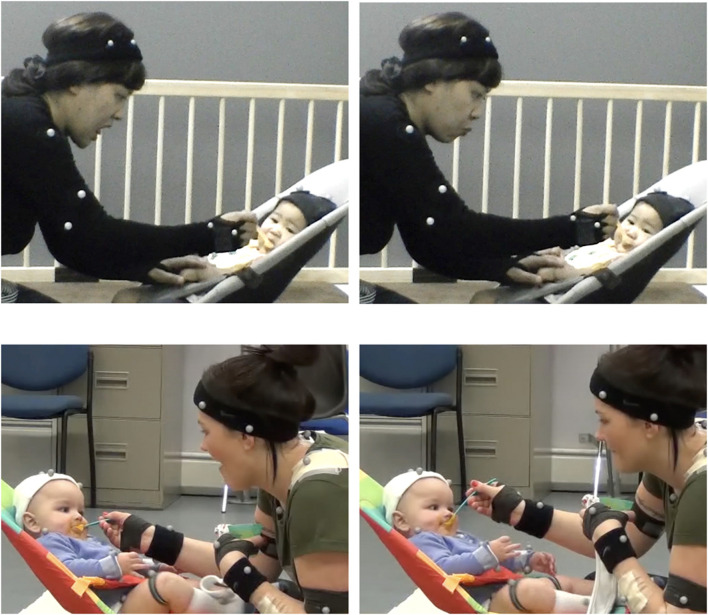
A Japanese (top) and a Scottish (bottom) mothers showing an empathetic mouth movement (opening and closing) while her infant is taking food.

As they feed their young infants, some mothers show an interesting behavior of mouth movement (Figure 1) which is isomorphic with the infants’ mouth movement (Negayama, 1993; Kawata, 2014; Toyama, 2014). The behavioral symmetry could be a demonstration of mother-infant intersubjectivity. The neurophysiological background of behavioral mirroring has recently been discussed (Meyza and Knapska, 2018), and mouth mirror neurons are found for ingestive behavior in non-human primates (Ferrari et al., 2003).

Co-occurrence of symmetrical mouth movements is a sign of empathy in the feeding mothers (Negayama, 1993), and complements infant-mother “sympathy” as directly feeling the other person’s affective and intentional state (Trevarthen, 2016). It is supposed that orofacial communicative behavior has its evolutionary origin in the mother–infant dyad (Ferrari and Coudé, 2018). Non-verbal expressive actions of the body serve to mediate connectedness, and together with the mirror neuron system enables ‘direct neural resonance,’ a specific neural mechanism of shared affective and intentional neuromotor states that afford informed, intersubjective interactions with others (Gallese, 2009; Gallagher, 2012).

Human movements are never just functional and performative, but simultaneously express feeling in their kinematic form (Stern, 2010). These ‘forms of vitality’ convey an affective quality within the functional, performative aspects of movement that enrich intersubjectivity, sharing feelings in the intentional acts (Di Cesare et al., 2017; Di Cesare et al., 2020). And withholding expectation by pausing or coming in too quickly can raise arousal to generate excitement, or anxiety (Gratier, 2003).

This feeling is shared between individuals in their form and timing of body movement, made in the intersubjective ‘dance’ of their interaction (Malloch and Trevarthen, 2009a). Sharing feelings in sympathy, individuals may then reflect on these empathically to give “emotional and mental sensitivity to another’s state” (de Waal and Preston, 2017). Where sympathy names feelings in direct resonance, empathy allows for additional reflection or mentalizing and “assessing the reasons for it and adopting the other’s point of view.” Sympathy allows the organism to quickly relate to the states of others, and effectively empathize with their experience. These are essential for the regulation of social interactions, coordinated activity, and cooperation toward shared goals (Clay et al., 2018). In this paper, we employ a mother-infant feeding paradigm as a lens with which to observe the nuance of the intersubjective relation between mother and infant. Mother-infant feeding requires shared timing and symmetry of behavior. It is a clear and accessible case of embodied mother-infant intersubjectivity, with the maternal empathetic mouth opening giving one sensitive behavioral index of it.

### Differences in Mother–Infant Intersubjectivity by Infant’s Age and Culture

Importantly, interactional synchrony is required between mothers and infants to attune and harmonize their behaviors for cooperative effect. The process of mother-infant feeding is a continuous adjustment of the timing of food giving (maternal) and intake (infant) actions. Empathetic mouth opening by the mothers to the infants’ mouth opening at the moment of infant eating is a situation typical in everyday feeding of shared timing between the two.

The intentional nature of all human action, evident from before birth, underpins shared action understanding (Delafield-Butt and Gangopadhyay, 2013; Trevarthen and Delafield-Butt, 2017) generated within embodied, enactive interactions (Trevarthen and Delafield-Butt, 2013; Fantasia et al., 2014). Thus, motor timing in interaction shared between mother and infant presents an empirical measure of the shared intersubjective exchange. Set within shared projects, such as during infant pick-up or during feeding, these single acts become serially organized to give narrative structure, a narrative arc, as each act unfolds over time, altogether achieving the shared goal of the project (Trevarthen and Delafield-Butt, 2013; Rossmanith et al., 2014; Delafield-Butt and Trevarthen, 2015).

Empathy of feelings shared in sympathy cumulatively works over the whole process of feeding. Communication between feeders and infants with control, request, rejection, and cooperation between them (Stevenson et al., 1990; Negayama, 1993; Toyama, 2014; Fries et al., 2017; Nonaka and Stoffregen, 2020) reflects biological and cultural fundamentals of mother-infant relationships and their development.

Feeding induces a stronger empathetic mouth opening in mothers when providing food on their own than when just watching the infant being fed by the father (Negayama, 2000). This finding suggests that synchrony is not just reflection of behavior but could be boosted by the mothers’ anticipation of infants’ food intake. Eating is a continuous narrative between mother and infant containing intention-reading and anticipation.

From as early as 2 months old, infants adapt their action to match imminent action intentions of their mother. For example, when being picked up from the floor, an infant will arch its back and raise its arms and stiffen in preparation for the new forces and requirements of being picked up (Reddy et al., 2013). The infant is prospectively aware of the imminent consequences of their mothers’ actions, revealing an anticipatory awareness and self-generated adaptive, preparatory response to meet those expected demands. This basic action understanding is the embodied basis of understanding other minds (Trevarthen, 2001; Gallagher, 2012).

As demonstrated in Japanese mother-infant tickling play (Ishijima and Negayama, 2013), infants start to show joint attention with the mothers to a part of the infant’s body at 6 months. This is “*proto-triadic* relationship” (Negayama, 2012, forthcoming) that precedes “*genuine triadic* relationship” at 9 months (Tomasello, 1995; Negayama, forthcoming). Feeding is an example of the triadic relationship between parent, infant and food. The development of this triadic relationship should be reflected in differences of behaviors and their timing.

Feeding is also an accessible, important paradigm to understand cultural differences in intersubjectivity in the mother–infant relationship. We are particularly interested in whether or not and how the mothers in Japan and Scotland differ in their empathetic mouth openings.

Previous studies have shown Japanese and Scottish mothers differ in distance regulation with their infants, and its manner (Negayama and Trevarthen, under review). Scottish mothers took greater initiative than Japanese mothers in this aspect. Japan–Scotland differences were also found in bedtime and sleep routines in both the home and day nurseries (Negayama, 1997; Negayama and Kawahara, 2010). Scottish mothers prefer putting children to bed without physical contact, whereas the Japanese mothers keep physical contact until their children fall asleep. The mothers and infants in Japan more often co-sleep at night, whereas those in the Western countries prefer to sleep separately (Caudill and Weinstein, 1969). These findings suggest a stronger empathy toward their children in Japanese mothers, and a preference for peaceful togetherness rather than their independence.

Interestingly, Japanese mothers often consume the food left by their infants, and give their own food to their infants when their infants’ plates are empty (“cross-feeding,” Negayama, 2006), which suggests they actively generate a sense of “oneness,” a significant intimacy of shared, common experience in the dyad. This behavior is seldom observed in Scotland. Scottish mothers quickly withdraw from the role of feeder when their infants become autonomous in eating (Negayama, 2000). In contrast, Japanese infants refuse to be fed passively and do not establish independence in eating until later (Negayama, 1993). These examples suggest that Japanese mothers are more strongly guided by their empathy and motivation for shared experience with their infants.

On the other hand, if mothers prefer to direct and control their infants, then their infant’s food intake would be regulated more strongly by their mothers. Conversely, if the mothers prefer to facilitate and share their infants’ experience, then the infants would become more autonomous and the mothers will follow the infants. Thus, the present study sought to shed light on mother–infant intersubjectivity in feeding and its cultural differences in Japan and Scotland, by measuring the timing of their feeding interactions, especially over mouth openings of mothers and infants. It also could be postulated that cultural differences in mother–infant feeding relationships are expected to be evident over development, in this case between 6 and 9 months of age.

### Purpose of the Present Study

Coordinated action timing and synchrony of feeding behaviors between mother and infant are for efficient feeding, matching the intentions of the mother and the infant. This matching is examined by recording the timing of the mouth openings of mother and infants during the feeds. Apparently ‘synchronous’ behaviors could be found to be asynchronous when examined in detail. For example, a mother may open her mouth earlier than her infant, or *vice versa*. Isomorphism of the two behaviors makes the minute analysis easy. The initiative for action in the intersubjectivity of mother-infant feeding can be examined by comparing the onset times of their mouth openings with micro-analysis of their occurrence. Alternatively, the mother’s mouth opening might be regulated by some adjacent behaviors, for example, by the spoon’s arrival (i.e., stagnation) at the infant’s mouth, and insertion into it.

Fine micro-analysis of interaction timing during feeding serves as a promising window for exploring the age and cultural differences in mother–infant relationships. The basic question here is: Is the mother’s empathetic mouth opening directly caused by the infant’s mouth opening, or elicited by the mother’s own intention? To answer this question, three behaviors of mother’s empathetic mouth opening, infant mouth opening, and spoon carriage (particularly spoon arrival and insertion) are analyzed and compared. The analysis may demonstrate a variation by age and culture in the manner and style of more general mother–infant intersubjectivity, specifically analyzed here during feeding.

## Materials and Methods

### Participants

Eleven Japanese (two boys and nine girls, five first-borns) and 10 healthy Scottish infants (five boys and five girls, seven first-borns) at the ages of 6 months participated with their mothers. They were recruited at local nursery schools by delivering an invitation letter in Japan and personally through word of mouth, parent groups, and nurseries in Scotland. Each mother and infant pair participated in the observation twice: first at the infants’ age of ca. 6 months (*Age 1*, mean ages/SDs were 192.0/9.1 and 191.6/24.1 days in Japan and Scotland, respectively); and second at ca. 9 months (*Age 2*, mean ages/SDs were 282.2/16.2 and 289.8/16.7 days in Japan and Scotland, respectively). The other details of the participants are provided in Negayama et al. (2015). Participant numbers were limited and considered acceptable within the design due to the study requiring a precise time of laboratory-based recording within limited developmental periods.

This study was approved by the Ethical Committee of Waseda University (No. 2012-273) and the University of Strathclyde Ethics Committee. Written informed consent was obtained from each mother or father at the start of the study.

### Procedure and Data Recording

The present study is a part of a larger research project carried out at *Ages 1* and *2*, in which (1) mothers put down then picked up their infants from the floor, (2) mothers fed their infant with solid food with a spoon, (3) mothers tickled their infant in free play for about 15 min, and (4) mothers and infants played an action-word game task. The first part (study of pick-up) has already been reported (Negayama et al., 2015).

Data recordings of Japanese and Scottish participants were carried out at a laboratory at Waseda University in Japan and another at the University of Strathclyde in Scotland. The entire process was recorded using two or more standard home video cameras with the assistance of a motion-capture system. The mothers were briefly interviewed after the experiment for background information on the infants (the birth date, family composition, parents’ education and occupation, *etc.*). Written informed consent was obtained from each mother or father.

One Japanese case and one Scottish case were not observed at *Age 1* because solid feeding was not started yet. Feeding of 10 Japanese and 9 Scottish mother-infant dyads was filmed at *Age 1*, and of 11 Japanese and 10 Scottish dyads was filmed again at *Age 2*. Mothers brought solid foods from home to the laboratory and gave them to their infants with a spoon in their own way. The entire session was videotaped in each observation.

### Behavioral Analysis

Feeding is a sequence of behaviors: food is scooped by a spoon and carried to an infant’s mouth. The spoon stagnates before (or after in rare cases) reaching the infant’s mouth (*arrival*), and is *inserted* (spoon tip passing the edge of the lips) into the infant’s mouth. If the spoon does not stagnate, the time of arrival equals to the time of insertion. The infant *opens* the mouth at the moment of spoon insertion, but the mouth opening may be later than the insertion time. Some mothers *coerce* the infant to take the food provided by an exaggerated shooting movement toward the infant’s mouth to elicit the infant’s mouth opening. The spoon can be inserted and *pulled out* repeatedly until there is no food left on the spoon (*final withdrawal*), and then the spoon is *returned* to the initial position. *One spoon* refers to feeding from the start of the spoon carriage to the return. Simply the initial five consecutive spoon feeds were analyzed for these behaviors.

During feeding, mothers exhibit *empathetic mouth opening* (sometimes with chewing and closing), a fluid and reflexive behavior without reflective mentalization or strong volition. Another type of mouth opening also occurred which was to intentionally encourage the infant to take the food. This type quite often occurred with vocalization. In the present study, we focused on the former mouth movement, which was usually silent, as a behavior indicative of the mother’s intersubjectivity to the infant and ignored the latter one.

For a fine analysis of mother-infant interactions concerning the mother’s empathetic mouth opening, a good video image of the mouth movements by the mothers and infants was needed. However, the initial five spoons were not appropriate for this analysis because the mother talked or the hand of the mother or infant hid their mouth. So, we selected as many as possible other episodes out of the entire feeding session for the analysis. the number of episodes collected by this way were 43 and 43 (range/median = 0–7/6 and 0–7/6) for Japanese *Age 1* and *Age 2*, respectively, and 50 and 52 (range/median = 3–5/5 and 3–9/4.5) for Scottish *Age 1* and *Age 2*, respectively.

The time sequence of the spoon-feeding episodes was measured using the video analysis software ELAN (Version 5.9.1) [Computer software] (2020), and then the time gaps of the behavior occurrences were calculated from the onset times of adjacent behaviors to determine the sequential relationship of the behaviors.

Because of the small numbers of samples and episodes, age and cultural comparisons were made on the basis of median values of the time for each dyad, and non-parametric tests were conducted for statistical analyses. Differences between the two age periods were examined by pairwise comparison by the Wilcoxon’s rank-sum test, and differences between Japan and Scotland at each age were examined by Mann–Whitney *U* test. For both, the significance level was set at 0.05.

## Results

In order to substantiate intersubjectivity by a behavior, the timing and shape should be shared. Adjustment of mutual behaviors is needed for feeding, which is measured by timing of onset. The time structure of the behaviors, the synchrony of the mouth openings of the mothers and infants measured by the onset time are the focus of the analysis. Firstly, to understand the characteristics of the feeding interactions, a sequence of feeding was analyzed. The onset time and duration of each behavior were compared between the two stages (*Age 1* and *Age 2*) and between the two countries (Japan and Scotland) in general based on both the initial five feeding episodes and the additionally selected episodes. Then secondly, the mother’s empathetic mouth opening was focused on, and the relationship of the timings of the behaviors of the mothers and infants were analyzed based on the additionally selected episodes.

### Sequence of Feeding

**FIGURE 2:**
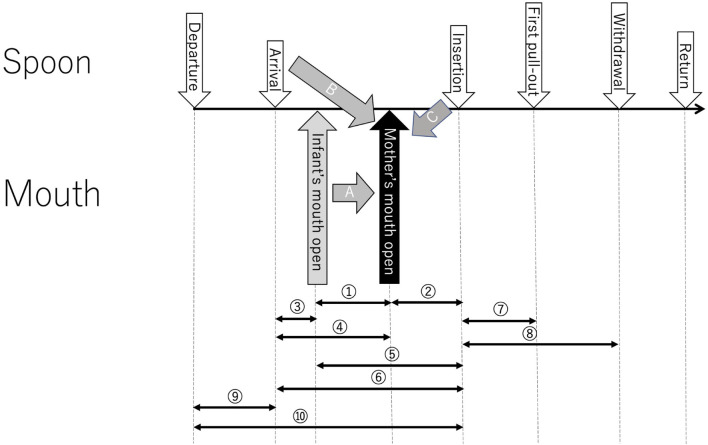
The sequence of spoon-feeding segmented by the onset time of behaviors. The ten intervals denoted by circled numbers were selected for analysis. Arrows A, B, and C are assumed causal directions of the mother’s empathetic mouth opening.

One spoon carriage consisted of a series of different behavioral components (Figure 2): spoon departure from the starting point, spoon arrival near the infant’s mouth, spoon insertion into the mouth, first spoon pulling-out, (repetition of insertion and pulling-out), final spoon withdrawal, and spoon return to the initial position. The infant’s mouth opening to capture food occurs typically somewhere between spoon arrival and insertion. The relationships of the mother’s empathetic mouth opening with the infant’s mouth opening (Arrow A), spoon arrival (Arrow B), and spoon insertion (Arrow C) were focused on.

A sequence of spoon movements can be divided into seven sections by the onset times of those behavioral components. Ten intervals were identified for analysis as indicated by Intervals ① to ➉ in Figure 2. The feeding sequence was normally composed of these sections, and a general time sequence of feeding was separately compared between the two age points and between the two countries on the basis of the lengths of the intervals in the initial five spoons (Table 1).

**TABLE 1:** Comparison of median behavior intervals (sec) by country and age.

	From	To		Age	Country		Mann–Whitney
					Japan	Scotland	
➈	Departure	Arrival		1	1.77	2.18	0.97
				2	1.44	1.99	0.01*
			Wilcoxon		0.037*	0.86	
➉	Departure	Insertion		1	3.15	3.87	0.40
				2	1.69	2.37	0.13
			Wilcoxon		0.009**	0.008**	
➇	Insertion	Withdrawal		1	4.09	3.07	0.28
				2	1.27	1.08	0.35
			Wilcoxon		0.009**	0.008**	
➆	Insertion	Pull-out		1	3.82	1.09	0.028*
				2	1.26	0.95	0.15
			Wilcoxon		0.007**	0.17	
➅	Arrival	Insertion		1	0.18	0.56	0.60
				2	0.45	0.02	0.56
			Wilcoxon		0.68	0.87	
➂	Arrival	Infant’s mouth open		1	0.09	0.27	0.50
				2	0.20	–0.06	0.47
			Wilcoxon		0.96	0.68	
➄	Infant’s mouth open	Insertion		1	0.32	0.24	0.66
				2	0.39	0.36	0.51
			Wilcoxon		0.39	0.31	
➀	Infant’s mouth open	Mother’s mouth open		1	0.11	0.14	0.30
				2	0.35	0.37	0.74
			Wilcoxon		0.26	0.86	
➃	Arrival	Mother’s mouth open		1	0.01	0.58	0.05
				2	0.40	0.42	0.80
			Wilcoxon		0.14	0.44	
➁	Mother’s mouth open	Insertion		1	0.04	0.01	0.22
				2	0.11	0.15	1.00
			Wilcoxon		0.44	0.26	

**p < 0.01, *p < 0.05.

*The bold means statistical significance.*

The mother’s empathetic mouth movement was the focus of the present study, but in Table 1, the behavior did not show significant differences between ages in Japan and Scotland (Wilcoxon’s rank-sum test) or between countries at *Age 1* as well as *Age 2* (Mann–Whitney *U* test) in any of the Intervals ① – ⑥. Statistical significance was found only in the Intervals ➆ – ➉, i.e., preceding and following behaviors sandwiching the interactions containing the mouth opening. Thus there were no age or cultural differences in the length of core interactions containing the mouth openings.

In Japan, spoon arrival time (calculated from the start of the spoon’s movement toward the infant, Interval ➈) and its insertion time (also calculated from the start of the spoon’s movement toward the infant, Interval ➉) were longer at *Age 1* than at *Age 2* (Wilcoxon’s rank-sum test, *p*’s < 0.05 and < 0.01, effect sizes = 0.66 and 0.82, *n* = 10, for ➈ and ➉, respectively). This means that Japanese mothers spent longer in the preparatory phase of feeding at *Age 1*. However, such an age difference was observed in Scotland only for spoon insertion (Wilcoxon’s rank-sum test, *p*’s = 0.86 and < 0.01, effect sizes = 0.06 and 0.89, *n* = 9, for ➈ and ➉, respectively).

The duration of spoon-in-the-mouth (calculated as the duration between insertion and first withdrawal, Interval ➆) was also longer at *Age 1* than at *Age 2* in Japan (Wilcoxon’s rank-sum test, *p* < 0.01, effect size = 0.85, *n* = 10), but not significantly different between ages in Scotland (Wilcoxon’s rank-sum test, *p* = 0.17, effect size = 0.45, *n* = 9). This means that Japanese mothers gave their infants more time for food intake at *Age 1*. The total time from spoon insertion to withdrawal (i.e., final pulling out, Interval ➇) was shorter at *Age 2* than at *Age 1* in both Japan and Scotland (Wilcoxon’s rank-sum test, *p* < 0.01, effect size = 0.82, *n* = 10), suggesting a more efficient and cooperative food-taking behavior on the side of the infants and smoother, more in-step food-giving on the side of the mothers at *Age 2*.

The Japan–Scotland comparison shows a significantly longer duration of the time of spoon-in-the-mouth until its withdrawal at *Age 1* (calculated as the duration of the spoon in the mouth from insertion until the first spoon pulling-out, Interval ➆, Mann–Whitney *U* = 18, *p* < 0.05, effect size = 0.51, *n* = 19), but not at *Age 2* in Japan. This indicates that Japanese mothers were more patient and appeared to wait for their young infants to take the food from the spoon when it was in the mouth than their Scottish counterparts with their 6-month-old infants. Conversely, a quicker carrying of a spoon (Interval ➈) was found in Japan than in Scotland at 9 months of age (Mann–Whitney *U* = 91, *p* = 0.01, effect size = 0.55, *n* = 21), but not at the earlier age. Despite their patience at the younger age, feeding times between Japanese mothers and infants became well-coordinated quickly.

**FIGURE 3:**
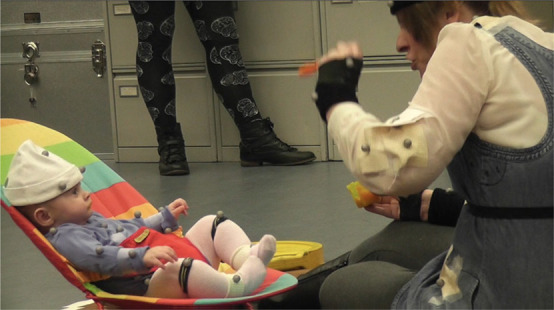
Coercive behavior in a Scottish mother. The mother performs a shooting movement of the spoon toward her infant.

**FIGURE 4:**
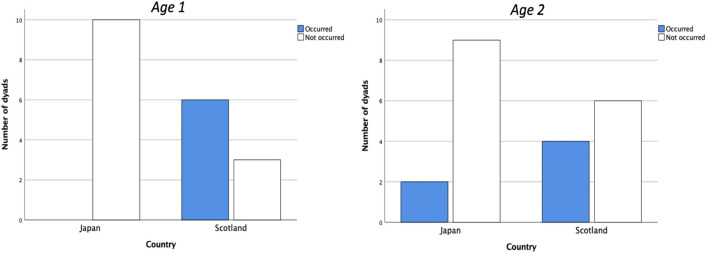
Coercive feeding in Japanese and the Scottish mothers at *Age 1* (left) and *Age 2* (right). Fisher’s exact test demonstrated significance between countries at *Age 1*, but not at *Age 2*. The differences in *Ages 1* and *2* are significant (*p* < 0.01) and not significant, respectively.

Coercive behavior (Figure 3) was seen in some mothers during the movement of the spoon toward the infant’s mouth (Interval ➈). Figure 4 shows the incidence of coercive feeding behavior in Japanese and Scottish mother-infant dyads at *Ages 1* and *2*. This behavior induced the infants to take the food, provided by an exaggerated shooting movement of the spoon approaching the infant’s mouth. It was significantly more frequent in Scottish mother-infant dyads at *Age 1* (Fisher’s exact test, *p* < 0.01), but not at *Age 2*. This appears to indicate greater initiative in feeding by Scottish mothers.

In contrast, Japanese mothers appeared inclined to adapt to the infants’ initiative, waiting for an adequate time for their infants to take the food. The spoon was then kept in the infants’ mouth for a significantly longer time in the Japanese dyads than in the Scottish ones at *Age 1* (Table 1). This suggests that the Japanese mothers have greater patience and a stronger infant-centeredness, allowing for and following their infants’ initiative for food intake.

### Relationship Between the Infants’ Mouth Opening and Their Mothers’ Empathetic Mouth Opening

The general analysis of the initial five spoons did not show any significant differences in the interactions relating with mothers’ empathetic mouth movement. So the behavior was further analyzed in detail by deliberately selected video episodes in which the mothers’ empathetic mouth opening was clearly observable.

This behavior might be induced by the infant’s mouth opening (Arrow A in Figure 2), the mother’s intention to insert the spoon into her infant’s mouth at the spoon arrival might trigger it (Arrow B), or the mother’s spoon insertion might evoke her mouth opening (Arrow C). Coupling the timing of the mother’s mouth opening with that of the infant’s and the spoon arrival/insertion gives a hint to understand the mechanism underlying the mother’s empathy.

**FIGURE 5:**
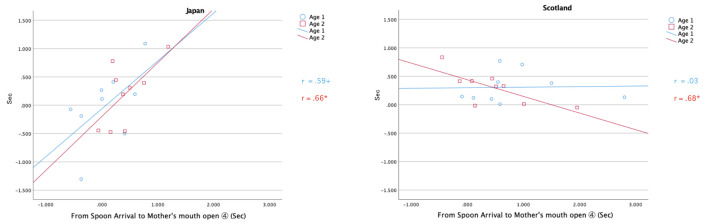
Time differences (Sec) in the onset of the infant’s mouth opening to that of mother’s mouth opening (Interval ①; y-axis) along the time from spoon arrival to the mother’s mouth opening (Interval ④; x-axis) at *Age 1* (blue) and *Age 2* (red) in Japan (left) and Scotland (right). *p < 0.05, ^+^*p* < 0.10.

Figure 5 is a scatter plot of the differences of the onset times between the infants’ mouth opening and that of the mothers’ empathetic mouth openings (Interval ➀; *y*-axis), along with the time interval from the spoon’s arrival to the mother’s mouth opening (Interval ④; *x*-axis) in Japan and Scotland. Data from *Age 1* (blue circles) and *Age 2* (red squares) are presented.

Time gaps between the Scottish infants’ and mothers’ mouth openings were more or less constant mostly within a 0–0.5 s range. The symbols were vertically plotted over zero for them, which means that the mothers quickly opened their mouths almost always later than the infants’ mouth opening. In comparison, the symbols for Japanese mothers scattered more widely around zero, showing that the Japanese dyads were more flexible and variable in their interaction.

The regression line at *Age 1* is horizontal in Scotland, meaning that the Scottish mothers’ mouth openings were a direct reaction to the infants’ preceding mouth opening irrespective of the length of time after spoon arrival. This is related to the mothers’ coerciveness in feeding. At *Age 2*, however, the time gap in Scotland decreased with the time after arrival. This reflects a change in the Scottish infants at *Age 2* to start opening their mouths even before the spoon arrival by reading their mothers’ intention.

In contrast, some Japanese mothers opened their mouths earlier than their infants’ mouth opening (the symbols plotted under zero). This means that Japanese mothers’ mouth openings were not a direct reaction to their infants’ behaviors, but were induced by the mother’s own anticipation or intention. It is notable that the time elapsed from the infant’s mouth opening to the mother’s mouth opening increased along with the length of time from spoon arrival at both *Ages*, which suggests that the Japanese mothers’ mouth opening was always linked with her own spoon carriage rather than evoked by the preceding infants’ mouth opening.

**FIGURE 6:**
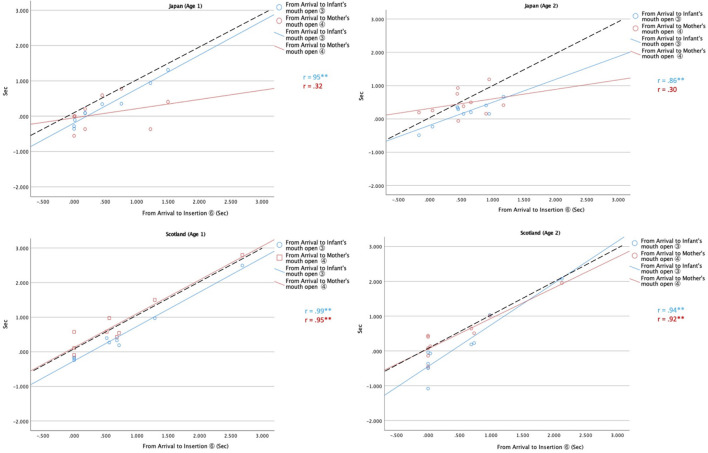
Time (Sec) of the mouth opening by the infants (blue) and the mothers (red) after the spoon arrival (Intervals ③ and ④; *y*-axis) along the time elapsed after the spoon arrival to insertion (Interval ⑥; *x*-axis). The dashed line indicates the time of spoon insertion after arrival. **p < 0.01.

Figure 6 compares the times of the infants’ mouth opening (blue circles) and the mothers’ mouth opening (red squares) after the moment of the spoon’s arrival distributed along the time elapsed from the spoon arrival to its insertion. The dashed line indicates the time of spoon insertion after arrival. If the mouth openings of the mothers and infants were induced by spoon insertion, then the lines would parallel the dashed line. When the regression lines are below the dashed line, it means that the mouth opened before spoon insertion. But if the occurrence is coupled with spoon arrival, the regression lines should be horizontal, near the zero level.

Naturally, the infants’ mouth opened near the time of spoon insertion, and the blue lines are almost parallel to the dashed line. The red lines are also parallel to and overlap the dashed line in Scotland, which means that the Scottish mothers showed perfect co-occurrence of spoon insertion and the infant’s and mother’s mouth openings.

On the other hand, Japanese mothers (red lines of the top two figures) showed a quite different tendency, i.e., being constantly horizontal at slightly above the zero level, which means that the Japanese mothers tended to open their mouths immediately following the spoon arrival irrespective of the time of the spoon insertion (proving Arrow B rather than A or C of Figure 2). In other words, the Japanese mothers apparently carried the spoon to the arrival point at just the right moment for the infants’ food intake judged from their infants’ state, and adjusted the spoon carriage to that state. The mothers stagnated the spoon and waited for the infant’s mouth opening if the infants unexpectedly did not show it immediately. This appears to be a more infant-centered feeding style that observes and imagines their infants’ state, anticipating their infant’s food taking. The mother’s mouth opening was thought to be caused by her empathetic motivation. In comparison, the Scottish mothers adapted the carriage of the spoon to the infants’ condition *after* arrival. Together with their high incidence of coercive feeding, this suggests a more assertive feeding style that attempts to lead the infants. This suggests their mouth opening was a response to the infant’s food-taking evoked by the mothers’ coercive spoon carriage (Arrow A rather than B or C of Figure 2).

The Scottish dyads were concentrated around the left-end zone of the horizontal axis. *That is*, little arrival-insertion gap, indicating that the spoon was pushed directly to the infant’s mouth without preceding stagnation or adjustment. This tendency can be pointed out in Scotland at both *Ages 1* and *2*, but is particularly remarkable at 9 months of age. The symbols of Japanese dyads were more widely scattered because the mothers tended to be more deliberate in attuning to their infant’s initiative before they initiated carriage of the spoon for feeding.

More constant reaction of the Scottish mothers to the infants’ mouth opening with a short interval mentioned above suggests that their intersubjectivity was directly triggered by the infants’ mouth opening. Intersubjectivity among the Japanese mothers, in contrast, appears to be based on empathy with the infants’ intentions. In other words, the Japanese mothers’ intersubjectivity was of a more anticipatory nature that followed their infant’s initiative, and the Scottish mothers’ intersubjectivity was of more directive.

The analyses above show the connection between the mouth openings of infants and mothers. To examine the regularity of the interactions containing the mouth openings of the mother and infants, the correlation matrix of Intervals ➀ to ➅ at *Age 1* and *Age 2* by Spearman’s rho are shown separately for Japanese and Scottish dyads in Table 2. Regularity demonstrated by high correlations between the intervals was thought as an evidence of structuredness of the feeding sequence, which made the mothers and infants to synchronize their mutual behaviors easier.

**TABLE 2:** Correlations of length of intervals at *Age 1* (Top) and *Age 2* (Bottom).

*Age 1*		From arrival to insertion ➅	From arrival to infant’s mouth open ➂	From infant’s mouth open to insertion ➄	From infant’s mouth open to mother’s mouth open ➀	From arrival to mother’s mouth open ➃	From mother’s mouth open to insertion ➁
Japan	From arrival to insertion ➅	1.000	0.976**	–0.399	–0.276	0.494	0.059
	From arrival to infant’s mouth open ➂		1.000	–0.345	–0.383	0.417	0.133
	From infant’s mouth open to insertion ➄			1.000	0.267	–0.250	0.117
	From infant’s mouth open to mother’s mouth open ➀				1.000	0.567	−0.750*
	From arrival to mother’s mouth open ➃					1.000	−0.783*
	From mother’s mouth open to insertion ➁						1.000
Scotland	From arrival to insertion ➅	1.000	0.831**	–0.051	–0.034	0.695*	–0.186
	From arrival to infant’s mouth open ➂		1.000	–0.150	–0.367	0.800**	0.017
	From infant’s mouth open to insertion ➄			1.000	0.017	–0.267	0.017
	From infant’s mouth open to mother’s mouth open ➀				1.000	0.183	−0.833**
	From arrival to mother’s mouth open ➃					1.000	–0.450
	From mother’s mouth open to insertion ➁						1.000

***Age 2***		**From arrival to insertion ➅**	**From arrival to infant’s mouth open ➂**	**From infant’s mouth open to insertion ➄**	**From infant’s mouth open to mother’s mouth open ➀**	**From arrival to mother’s mouth open ➃**	**From mother’s mouth open to insertion ➁**

Japan	From arrival to insertion ➅	1.000	0.655*	0.309	–0.455	0.224	0.564
	From arrival to infant’s mouth open ➂		1.000	–0.309	–0.624	0.115	0.527
	From infant’s mouth open to insertion ➄			1.000	0.188	0.345	–0.006
	From infant’s mouth open to mother’s mouth open ➀				1.000	0.539	−0.915**
	From arrival to mother’s mouth open ➃					1.000	–0.479
	From mother’s mouth open to insertion ➁						1.000
Scotland	From arrival to insertion ➅	1.000	0.899**	−0.795**	−0.860**	0.847**	–0.317
	From arrival to infant’s mouth open ➂		1.000	−0.879**	−0.758*	0.964**	–0.600
	From infant’s mouth open to insertion ➄			1.000	0.806**	−0.794**	0.345
	From infant’s mouth open to mother’s mouth open ➀				1.000	−0.648*	0.212
	From arrival to mother’s mouth open ➃					1.000	–0.564
	From mother’s mouth open to insertion ➁						1.000

**p < 0.01, *p < 0.05.

*The bold means statistical significance.*

The most conspicuous result is the high ratio of significant correlations among the intervals at 9 months in Scotland. Particularly, there was an increase of significant correlations in the combinations with the infants’ mouth opening in Scottish dyads compared to Japanese ones. This means that the time series of behaviors was more structured and therefore predictable in Scotland at *Age 2*.

We interpret this higher correlation as a result of reduced lability on the infant behavior due to the coercive maternal feeding style. This gave a compelling, structured interaction. Thus, the Scottish infants were unable to adjust their behaviors to this feeding style at *Age 1*, and later came to adjust their behavior to the mothers’ feeding at *Age 2*.

In contrast, the Japanese mothers allowed greater freedom for their infants, which was possible by the more sensitively attuned feeding by the mothers that followed their infants and made the interaction more flexible. In Japan, the interactions became more complex, because the infants’ spontaneity was prioritized and thereby interactions were more diverse. Thus the correlation of the Japanese dyads remained low at *Age 2*.

## Discussion

The results indicated that Japanese mothers were more attuned to their infants’ states during feeding when the infants were at *Age 1*. In contrast, the Scottish mothers were more assertive in the spoon carriage with displays of coercive behavior with their 6-month-old infants. The mothers of both countries frequently opened their mouths when their infants’ mouths opened to take food. The Scottish mothers’ mouth openings occurred as a response to the infants’ mouth opening by the spoon insertion, which itself was initiated by their more directive, mother-centered style of feeding. In contrast, Japanese mothers opened their mouths by empathic anticipation, encouraging their infants’ food-taking behavior based on a more affective infant-centeredness.

### Synchrony and Intersubjectivity

Temporal coordination in the mother-infant interaction during feeding is guaranteed by the infants’ timely mouth opening at the moment of spoon insertion and the mothers’ adjustment of their behavior to the infants’ states.

The infants opened their mouths almost simultaneously but slightly earlier than the spoon insertion. This behavior was possible because of the infants’ anticipation of the insertion, to which Tau Theory might be applicable for an explanation of the perception of gap-closure (Lee, 1976; Lee, 2009) operative in infant perception and action (Agyei et al., 2016; Delafield-Butt et al., 2018). This could be an underlying mechanism of the intersubjectivity between infants and mothers.

The infants responsively adjusted their mouth behavior to the mothers’ spoon carriage. Mothers also carried the spoon at the right moment for coordinated action and common purpose with their infants, which requires the mother to perceive her infant’s state of readiness, interest, and intention correctly. As the present study demonstrates, mothers open their mouths to their infants’ food intake. This mother–infant symmetry of behaviors is strong evidence of their intersubjectivity. The mothers gave food to the infants, but simultaneously behave as if they were being fed as well, which shows shared feeling. Mothers’ mouth opening occurred slightly later than the mouth opening of their infants, a feature more evident in Scotland.

Maternal experience of mother–infant intersubjectivity is different from, but deeply related to their infant’s experience of that same intersubjectivity (Trevarthen, 2001). Food-providing by mothers is a uniquely human behavior, allowing the mothers’ intersubjectivity and the infants’ intersubjectivity to intersect in a clear and explicit manner in body movement and overt behavior. According to Trevarthen and Hubley (1978) and Trevarthen (1998), infants acquire secondary intersubjectivity at 9 months, characterized by a genuine triadic relationship (Tomasello, 1995; Negayama, forthcoming). The well-coordinated feeding of the Scottish mothers and infants at 9 months despite the mothers’ coercive feeding at 6 months as well as the Japanese infants’ quicker food taking at 9 months than at 6 months can be explained by the developing attunement of the 9-month-old infants to the shared object, the spoon.

Co-occurrence of the same mouth openings (mirroring) in mothers and infants is another unique characteristic of human feeding. The synchronous mouth opening by a feeder was also observed in an infant’s father and a 2-year-old sibling at feeding infant (Negayama, 2006). Joint attention of the mother and the infant to an object in a triadic relationship requires taking the other’s perspective (Tomasello et al., 1993). This allows for infant learning the particular mindedness of his or her mother. Learning such perspective-taking is embodied within the intersubjective engagement between the mother and infant.

Tickling play is another interaction between mother and infant with joint attention to a part of the infant’s body, which similarly demonstrates this *proto-triadic* relationship evident at 6 months (Negayama, 2012). Feeding is another special, preliminary type of triadic relationship between mothers, infants, and food as a target. Food stimulates the infants’ naso-oral sense organs, but it also arouses the mothers’ taste and tactile naso-oral sensations. This is another dimension of mother-infant empathy, enriching the intersubjective triadic relationship with imagined expectations and real sensory experiences of taste, naso-oral sensations and sensorimotor tasks. And mother and infant ultimately share in the pleasure or displeasure in the accomplishment of the shared project (Delafield-Butt, 2018). This resonant relationship of intercorporeality can be understood as “embodied simulation” (Gallese, 2009), and the embodied triadic relationship is called a *quasi-triadic* relationship (Negayama, forthcoming; Negayama and Nakano, in press). Feeding shares the same basis of learning in co-created, co-operative projects that form the foundation of shared meaning-making invariant in human life (Trevarthen and Delafield-Butt, 2015; Delafield-Butt and Trevarthen, 2020).

### Age and Cultural Differences

The Scottish mothers appeared more coercive and took the initiative to provide food at 6 months of age, and their infants followed them. Furthermore, their tendency to push a spoon into the infant’s mouth without a preceding stagnation or adjustment sensitive to their infant’s intention was observed. The Scottish mothers were more mother-centered, and the infants adjusted their behavior to their mothers. This means that the process was framed by the mothers’ leadership, and the structure became simpler because of the infants’ compliance.

In contrast, there was a greater flexibility in Japanese mothers, which was related to their long and deliberate spoon carriage by their infant-centeredness and cooperativeness. The Japanese mothers in the infant pick-up experiment of this study were also gentler and more deliberate with kneeling and had a slower approach before picking up the infants than the Scottish mothers, and the Scottish mothers were quicker in crouching to pick up the infants than the Japanese mothers (Negayama et al., 2015). Therefore, the Japanese mothers’ intersubjectivity was framed by expectation of the infants’ food-taking rather than mirroring of the infants’ behavior.

The Japanese mothers’ mouth opened with a constant length of time after arrival, irrespective of the time to insertion. In other words, the mothers started the carriage at an appropriate time for food intake and did not need to adjust the movement to the infant state after departure, because the two were already aligned: the Japanese infants were more autonomous than the Scottish infants, and the Japanese mothers followed their infants and waited for the right time to bring the spoon of food for feeding by monitoring them.

It is also notable that some Japanese mothers opened their mouths earlier than the infants’ mouth opening. These could be signs of anticipatory intersubjectivity among the Japanese dyads, and the infants would gradually learn the same psychological trait of intention-reading through the above-mentioned resonant experience between mother and infant at 6 months of age.

Such empathy-driven intersubjectivity is more likely to characterize Japanese dyads with infant-centeredness in their mothers. Japanese mothers’ strong empathy was also pointed out when putting the infants to sleep with physical contact. This was to avoid a sadness or hardship on both sides by being left alone (Negayama, 1997), which could be related to ‘*Amae*’ (Doi, 1992). In Scotland, on the other hand, the mothers’ strong coerciveness during feeding played an important role in its shared timing or synchrony. This is similar with Scottish mothers’ preference for isolated sleeping in a bed separate from their infants (Negayama, 2006, forthcoming). The Scottish mothers then mirrored their infants’ mouth opening, generating a reactive intersubjectivity. At 9 months, i.e., the age of secondary intersubjectivity and genuine triadic relationship, however, the Scottish infants became to open their mouth before the spoon insertion by reading the mother’s intention.

Intersubjectivity is the basis of coordination of feelings, intentions, and desires between bodies (Trevarthen, 2001). This coordination of movement and voice develops a shared project, or narrative, that allows for the development of inter-personal and cultural meaning (Gratier and Trevarthen, 2008; Malloch and Trevarthen, 2009b; Trevarthen and Delafield-Butt, 2013; Delafield-Butt and Trevarthen, 2015; Delafield-Butt, 2018). Mother-infant feeding timing and synchronization of mouth movements serve as indicators, or physical expressions of an embodied intersubjective resonance between them, serving to structure and organize the shared project. The different elements of timing, anticipation, and empathetic mirroring underpin the process. In this paper, we have identified differences in these psycho-motor structures that illustrate two different cultural types of intersubjectivity in Japan and Scotland, and suggested how these become culturally transmitted, and learned by the next generation.

Intentions are carried in movement, and the source of those intentions (empathetic or directive, as we have found here) becomes apparent in their shared experience. This allows learning of the particular maternal characteristics of mind-mindedness (Meins et al., 2002), and the transmission of a culture. Murray et al. (2018) postulate neurofunctional architecture which increases an occurrence of infant behavior when a caregiver mirrors the infant behavior. This functional architecture is a part of intersubjectivity of infants to respond in certain ways to specific forms of parental display (Murray et al., 2016). Bozicevic et al. (2021) demonstrate that cultural difference in maternal responsiveness mediate difference in the infant’s later communicative behavior.

Two different types of parenting have been repeatedly pointed out in contexts other than feeding: control or regulator type and warmth or facilitator type (Raphael-Leff, 1993; Azuma, 1994; Bornstein, 2015). These studies show Japanese parenting can be classified as the latter type, relying more on affective ties and empathy than parental control. On the other hand, Western parenting can be relatively classified as the former type. Our present result follows this dichotomy, and the different characteristics of intersubjectivity in feeding between Japan and Scotland described here are embodied in it. Japanese mothers’ lack of happiness and satisfaction in their motherhood compared to French and American mothers (Negayama et al., 2012) could be related to this limited autonomy in mothers.

### Study Limitations and Future Work

First of all, this study is based on a small sample size, and the findings should hence be taken with caution despite an availability of other studies providing support for the current conclusion. Non-parametric tests applied to the small sample size have limited what we could determine. It should also be noted that this study took place in semi-naturalistic conditions within a public, laboratory setting in front of video and motion capture cameras. Although the mothers were instructed to behave normally, they were on stage in public, and this may have affected their intersubjective posture with their infants. The performance we observed may have been a publicly acceptable form of interaction that might have differed from that in private. Future studies will confirm these results with unobtrusive video. Recent advances in markerless motion capture technology for more naturalistic data collection could test for potential differences between public and private forms of intersubjective cooperation in feeding, and in other shared behaviors.

### Further Perspectives

Eating is not just an activity where children take food to their mouths and swallow it. Instead, it is a cluster of behaviors related to parent-child relationships in a broader context. Feeding is a social activity involving both cooperation and antagonism between mothers and infants. Infants’ desires about what, when, and how they eat do not always accord with the expectations of their mothers. Parents and infants sometimes conflict with each other and try to read each other’s intentions and negotiate. The onset of refusal behavior by children in the transition from dependent to independent eating is a meaningful change (Negayama, 2011). This happens at about 9 months of age, the beginning of the genuine triadic relationship (Negayama, 1993).

Children trying to eat in the way they want and rejecting their parents’ control frustrates their parents who want to feed them properly. The children read the parents’ intentions and try to manipulate them. This is related to parents’ pushy feeding or coercive control, showing the conflicting nature of eating as a situation of mutual manipulation by the parents and the children (Jansen et al., 2017).

Through such negotiation and co-regulation, children establish a parent-child centrifugalism, promoting independence from their parents. It is not a coincidence that these changes co-occur with the development of secondary intersubjectivity at 9 months of age. Kawata (2014) explained this kind of children’s protest for autonomy in eating as “psychological reactance” (Brehm and Brehm, 2013). Companionship between mother and infant develops on the basis of this co-regulation (Trevarthen, 2006).

## Data Availability Statement

The raw data supporting the conclusions of this article will be made available by the authors, without undue reservation.

## Ethics Statement

The studies involving human participants were reviewed and approved by the Ethical Committee of Waseda University (No. 2012-273) and the University of Strathclyde. Written informed consent to participate in this study was provided by the participants, in the case of adults, and the participants’ legal guardian/next of kin, in the case of the infants. Written informed consent was obtained from the individual(s) and minor(s)’ legal guardian/next of kin for the publication of any potentially identifiable images or data included in this article.

## Author Contributions

KN contributed to the conception and design of the study, led data collection in Japan, and wrote the first draft of the manuscript. JD-B contributed to the design of the study, led data collection in Scotland, and co-authored and edited the manuscript. All authors contributed to methodology development and data collection. KN and KM performed the statistical analysis. All authors contributed to observation and manuscript revision, read, and approved the submitted version.

## Conflict of Interest

The authors declare that the research was conducted in the absence of any commercial or financial relationships that could be construed as a potential conflict of interest.

## Publisher’s Note

All claims expressed in this article are solely those of the authors and do not necessarily represent those of their affiliated organizations, or those of the publisher, the editors and the reviewers. Any product that may be evaluated in this article, or claim that may be made by its manufacturer, is not guaranteed or endorsed by the publisher.

## References

[B1] Agyei S. B. van der Weel F. R. R. van der Meer A. L. H. (2016). Development of visual motion perception for prospective control: brain and behavioral studies in infants. *Front. Psychol.* 7:100. 10.3389/fpsyg.2016.00100 26903908 PMC4746292

[B2] Azuma H. (1994). *Education and Socialization in Japan: A Comparison Between Japan and the United States.* Tokyo: University of Tokyo Press.

[B3] Bornstein M. H. (2015). “Children’s parents,” in *Handbook of Child Psychology and Developmental Science*, 7th Edn, eds Bornstein M. H. Leventhal T. (New Jersey NJ: Wiley), 55–132.

[B4] Bozicevic L. De Pascalis L. Montirosso R. Ferrari P. F. Giusti L. Cooper P. J. (2021). Sculpting culture: early maternal responsiveness and child emotion regulation – a UK-Italy comparison. *J. Cross-Cultural Psychol.* 52 22–42. 10.1177/0022022120971353

[B5] Brehm S. S. Brehm J. W. (2013). *Psychological Reactance: A Theory of Freedom and Control.* New York, NY: Academic Press.

[B6] Caudill W. Weinstein H. (1969). Maternal care and infant behavior in Japan and America. *Psychiatry* 32 12–43. 10.1080/00332747.1969.11023572 5779087

[B7] Clay Z. Palagi E. de Waal F. B. M. (2018). “Ethological approaches to empathy in primates,” in *Neuronal Correlates of Empathy: From Rodent to Human*, eds Meyza K. Z. Knapska E. (London: Academic Press), 53–66. 10.1016/B978-0-12-805397-3.00005-X

[B8] Cole N. C. Musaad S. M. Lee S.-Y. Donovana S. M. The S. T. R. O. N. G., and Kids Team. (2018). Home feeding environment and picky eating behavior in preschool-aged children: a prospective analysis. *Eat. Behav.* 30 76–82. 10.1016/j.eatbeh.2018.06.003 29894927

[B9] Connolly K. Dalgleish M. (1989). The emergence of a tool-using skill in infancy. *Dev. Psychol.* 25 894–912. 10.1037/0012-1649.25.6.894

[B10] de Waal F. B. M. Preston S. D. (2017). Mammalian empathy: behavioural manifestations and neural basis. *Nat. Rev. Neurosci.* 18 498–509. 10.1038/nrn.2017.72 28655877

[B11] Delafield-Butt J. (2018). “The emotional and embodied nature of human understanding: sharing narratives of meaning,” in *The Child’s Curriculum: Working with the Natural Voices of Young Children*, eds Trevarthen C. Delafield-Butt J. Dunlop A.-W. (Oxford: Oxford University Press). 10.1093/oso/9780198747109.003.0004

[B12] Delafield-Butt J. Trevarthen C. (2020). “Infant intentions: learning with others,” in *Encyclopedia of Teacher Education*, ed. Peter M. (Singapore: Springer Nature). 10.1007/978-981-13-1179-6_74-1

[B13] Delafield-Butt J. T. Freer Y. Perkins J. Skulina D. Schögler B. Lee D. N. (2018). Prospective organization of neonatal arm movements: a motor foundation of embodied agency, disrupted in premature birth. *Dev. Sci.* 21:e12693. 10.1111/desc.12693 29920860 PMC6220947

[B14] Delafield-Butt J. T. Gangopadhyay N. (2013). Sensorimotor intentionality: the origins of intentionality in prospective agent action. *Dev. Rev.* 33 399–425. 10.1016/j.dr.2013.09.001

[B15] Delafield-Butt J. T. Trevarthen C. (2015). The ontogenesis of narrative: from moving to meaning. *Front. Psychol.* 6:01157. 10.3389/fpsyg.2015.01157 26388789 PMC4557105

[B16] Di Cesare G. De Stefani E. Gentilucci M. De Marco D. (2017). Vitality forms expressed by others modulate our own motor response: a kinematic study. *Front. Hum. Neurosci.* 11:565. 10.3389/fnhum.2017.00565 29204114 PMC5698685

[B17] Di Cesare G. Gerbella M. Rizzolatti G. (2020). The neural bases of vitality forms. *Natl. Sci. Rev.* 7 202–213. 10.1093/nsr/nwz187 34692032 PMC8288905

[B18] Doi T. (1992). On the concept of Amae. *Infant Mental Health J.* 13 7–11. 10.1002/1097-0355(199221)13:1<7::AID-IMHJ2280130103>3.0.CO;2-E

[B19] ELAN (Version 5.9.1) [Computer software] (2020). *Nijmegen**: Max Planck Institute for Psycholinguistics, The Language Archive.* Available online at https://archive.mpi.nl/tla/elan (accessed March 30, 2020).

[B20] Fantasia V. De Jaegher H. Fasulo A. (2014). We can work it out: an enactive look at cooperation. *Front. Psychol.* 5:874. 10.3389/fpsyg.2014.00874 25152745 PMC4126490

[B21] Feldman R. (2003). Infant-mother and infant-father synchrony: the co-regulation of positive arousal. *Infant Mental Health J.* 24 1–23. 10.1002/imhj.10041

[B22] Feldman R. (2007). Parent–infant synchrony and the construction of shared timing: physiological precursors, developmental outcomes, and risk conditions. *J. Child Psychol. Psychiatry* 48 329–354. 10.1111/j.1469-7610.2006.01701.x 17355401

[B23] Ferrari P. F. Coudé G. (2018). “Mirror neurons, embodied emotions, and empathy,” in *Neuronal Correlates of Empathy: From Rodent to Human*, eds Meyza K. Z. Knapska E. (London: Academic Press), 67–77. 10.1016/B978-0-12-805397-3.00006-1

[B24] Ferrari P. F. Gallese V. Rizzolatti G. Fogassi L. (2003). Mirror neurons responding to the observation of ingestive and communicative mouth actions in the monkey ventral premotor cortex. *Eur. J. Neurosci.* 17 1703–1714. 10.1046/j.1460-9568.2003.02601.x 12752388

[B25] Fries L. R. Martin N. van der Horst K. (2017). Parent-child mealtime interactions associated with toddlers’ refusals of novel and familiar foods. *Physiol. Behav.* 176 93–100. 10.1016/j.physbeh.2017.03.001 28315360

[B26] Gallagher S. (2012). “Neurons, neonates, and narrative: from embodied resonance to empathic understanding,” in *Moving Ourselves: Bodily Motion and Emotion in the Making of Intersubjectivity and Consciousness*, eds Foolen A. Lüdtke U. Zlatev J. Racine T. (Amsterdam: John Benjamins). 10.1075/ceb.6.07gal

[B27] Gallese V. (2009). Mirror neurons, embodied simulation, and the neural basis of social identification. *Psychoanal. Dial.* 19 519–536. 10.1080/10481880903231910

[B28] Gratier M. (2003). Expressive timing and interactional synchrony between mothers and infants: cultural similarities, cultural differences, and the immigration experience. *Cogn. Dev.* 18 533–554. 10.1016/j.cogdev.2003.09.009

[B29] Gratier M. Trevarthen C. (2008). Musical narratives and motives for culture in mother-infant vocal interaction. *J. Consciousness Stud.* 15 122–158.

[B30] Ishijima K. Negayama K. (2013). Mother-infant interaction in tickling play: intention-reading based on narrative sharing. *Japanese J. Dev. Psychol.* 24 326–336.

[B31] Jansen P. W. de Barse L. M. Jaddoe V. W. V. Verhulst F. C. Franco O. H. Tiemeier H. (2017). Bi-directional associations between child fussy eating and parents’ pressure to eat: who influences whom? *Physiol. Behav.* 176 101–106. 10.1016/j.physbeh.2017.02.015 28215424 PMC5436628

[B32] Kawahara N. (2005). Development of eating with tool in toddlers. *Japanese J. Appl. Psychol.* 31 98–112.

[B33] Kawata M. (2014). *Primordium Mechanism of the Development of Self in Infancy: Origin of Objective Self and a Hinge Effect of Triadic Relationship.* Kyoto: Nakanishiya Shuppan.

[B34] Lee D. N. (1976). A theory of visual control of braking based on information about time-to-collision. *Perception* 5 437–459. 10.1068/p050437 1005020

[B35] Lee D. N. (2009). General Tau theory: evolution to date. *Perception* 38 837–850. 10.1068/pmklee 19806967

[B36] Malloch S. Trevarthen C. (eds) (2009a). *Communicative Musicality: Exploring the Basis of Human Companionship.* Oxford: Oxford University Press.

[B37] Malloch S. Trevarthen C. (2009b). “Musicality: communicating the vitality and interests of life,” in *Communicative Musicality: Exploring the Basis of Human Companionship*, eds Malloch S. Trevarthen C. (Oxford: Oxford University Press), 1–12.

[B38] Meins E. Fernyhough C. Wainwright R. Das Gupta M. Fradley E. Tuckey M. (2002). Maternal mind–mindedness and attachment security as predictors of theory of mind understanding. *Child Dev.* 73 1715–1726. 10.1111/1467-8624.00501 12487489

[B39] Meyza K. Z. Knapska E. (2018). *Neuronal Correlates of Empathy: From Rodent to Human.* London: Academic Press.

[B40] Murray L. Bozicevic L. Ferrari P. F. Vaillancourt K. Dalton L. Goodacre T. (2018). The effects of maternal mirroring on the development of infant social expressiveness: the case of infant cleft lip. *Hindawi Neural Plasticity* 2018 1–10. 10.1155/2018/5314657 30647731 PMC6311812

[B41] Murray L. De Pascalis L. Bozicevic L. Hawkins L. Sclafani V. Ferrari P. F. (2016). The functional architecture of mother-infant communication, and the development of infant social expressiveness in the first two months. *Sci. Rep.* 6:39019. 10.1038/srep39019 27966659 PMC5155249

[B42] Negayama K. (1993). Weaning in Japan: a longitudinal study of mother and child behaviours during milk- and solid-feeding. *Early Dev. Parent.* 2 29–37. 10.1002/edp.2430020106

[B43] Negayama K. (1996). “Eating behaviour up to weaning,” in *Eating*, eds Nakajima Y. Imada S. (Tokyo: Asakura shoten).

[B44] Negayama K. (1997). “Parent-child relationship and independence: focusing on Japan-U.K. comparison,” in *Cultural Psychology: Theory and proof*, eds Kitayama K. K. Azuma S. H. (Tokyo: Tokyo University Press), 160–179.

[B45] Negayama K. (2000). Feeding as a communication between mother and infant in Japan and Scotland. *Ann. Rep. Res. Clin. Center Child Dev.* 22 59–68.

[B46] Negayama K. (2006). *Child-care as Kowakare.* Tokyo: NHK Books.

[B47] Negayama K. (2011). Kowakare: a new perspective on the development of early mother-offspring relationship. *Int. Psychol. Behav. Sci.* 45 85–99. 10.1007/s12124-010-9148-1 21161454 PMC3043264

[B48] Negayama K. (2012). “Bodily contact as the basis of interindividual relationship,” in *The Basis of Development: Body, Cognition and Emotion*, eds Negayama K. Naka M. (Tokyo: Shin-Yo-Sha), 119–130.

[B49] Negayama K. (forthcoming). *Parent-infant Centrifugalism and Centripetalism: Overcoming the Prison of “Parenting”.* Tokyo: Waseda University Press.

[B50] Negayama K. Delafield-Butt J. T. Momose K. Ishijima K. Kawahara N. Lux E. J. (2015). Embodied intersubjective engagement in mother–infant tactile communication: a cross-cultural study of Japanese and Scottish mother–infant behaviors during infant pick-up. *Front. Psychol.* 6:66. 10.3389/fpsyg.2015.00066 25774139 PMC4342882

[B51] Negayama K. Kawahara N. (2010). Cross-cultural comparison of nursery staff’s tactics to put children into sleep between Japan and Scotland. *Japan J. Med. Psychol. Study Infants* 19 117–123.

[B52] Negayama K. Nakano S. (in press). “Co-players in companionship,” in *Rhythm, Sympathy, and Human Being: Celebrating the Rhythms, Sympathies, and Many Beings of Colwyn Trevarthen*, eds Delafield-Butt J. Reddy V. (Oxford: Oxford University Press).

[B53] Negayama K. Norimatsu H. Barratt M. Jean-François B. (2012). Japan–France–US comparison of infant weaning from mother’s viewpoint. *J. Reprod. Infant Psychol.* 30 77–91. 10.1080/02646838.2011.649473 22745518 PMC3379788

[B54] Negayama K. Trevarthen C. (under review). Mother-infant co-regulation of distance across age and culture. 10.1016/j.infbeh.2022.101741 35779387

[B55] Nonaka T. Stoffregen T. A. (2020). Social interaction in the emergence of toddler’s mealtime spoon use. *Dev. Psychobiol.* 62 1124–1133. 10.1002/dev.21978 32383216

[B56] Norimatsu H. (1993). Development of child autonomy in eating and toilet training: one-to three-year-old Japanese and French children. *Early Dev. Parent.* 2 39–50. 10.1002/edp.2430020107

[B57] Pliner P. (1994). Development of measures of food neophobia in children. *Appetite* 23 147–163. 10.1006/appe.1994.1043 7864609

[B58] Raphael-Leff J. (1993). Facilitators and regulators: two approaches to mothering. *Br. J. Med. Psychol.* 56 379–390. 10.1111/j.2044-8341.1983.tb01571.x 6661409

[B59] Reddy V. Markova G. Wallot S. (2013). Anticipatory adjustments to being picked up in infancy. *PLoS One* 8:e65289. 10.1371/journal.pone.0065289 23840324 PMC3688725

[B60] Rossmanith N. Costall A. Reichelt A. F. López B. Reddy V. (2014). Jointly structuring triadic spaces of meaning and action: book sharing from 3 months on. *Front. Psychol.* 5:1390. 10.3389/fpsyg.2014.01390 25540629 PMC4261719

[B61] Stern D. N. (2010). *Forms of Vitality.* Oxford: Oxford University Press. 10.1093/med:psych/9780199586066.001.0001

[B62] Stevenson M. B. Roach M. A. Ver Hoeve J. N. Leavitt L. A. (1990). Rhythms in the dialogue of infant feeding: preterm and term infants. *Infant Behav. Dev.* 13 51–70. 10.1016/0163-6383(90)90005-S

[B63] Tomasello M. (1995). “Joint attention as social cognition,” in *Joint Attention: Its Origins and Role in Development*, eds Moore C. Dunham P. J. (Hillsdale, NJ: Laurence Erlbaum Associates), 103–130.

[B64] Tomasello M. Kruger A. C. Ratner H. H. (1993). Cultural learning. *Behav. Brain Sci.* 16 495–552. 10.1017/S0140525X0003123X

[B65] Tovar A. Vaughn A. E. Fallon M. Hennessy E. Burney R. Østbye T. (2016). Providers’ response to child eating behaviors: a direct observation study. *Appetite* 105 534–541. 10.1016/j.appet.2016.06.020 27328098 PMC5067159

[B66] Toyama N. (2014). The development of Japanese mother–infant feeding interactions during the weaning period. *Infant Behav. Dev.* 37 203–215. 10.1016/j.infbeh.2014.01.002 24607573

[B67] Trevarthen C. (1998). “The concept and foundations of infant intersubjectivity,” in *Intersubjective Communication and Emotion in Early Ontogeny*, ed. Bråten S. (Cambridge: Cambridge University Press), 15–46.

[B68] Trevarthen C. (2001). Infant intersubjectivity: research, theory, and clinical applications. *J. Child Psychol. Psychiatry* 42 3–48. 10.1111/1469-7610.00701 11205623

[B69] Trevarthen C. (2006). “Stepping away from the mirror: pride and shame in adventures of companionship,” in *Attachment and Bonding: A New Synthesis (Dahlem Workshop Reports)*, eds Sue Carter C. Ahnert L. Grossmann K. E. Hrdy S. B. Lamb M. E. Porges S. W. (Cambridge: MIT Press), 55–84.

[B70] Trevarthen C. (2016). The spiritual nature of the infant self: an imaginative actor in relations of affection. *J. Consciousness Stud.* 23 258–282.

[B71] Trevarthen C. Aitken K. J. Vandekerckhove M. Delafield-Butt J. Nagy E. (2006). “Collaborative regulations of vitality in early childhood: stress in intimate relationships and postnatal psychopathology,” in *Developmental Psychopathology*, eds Cicchetti D. Cohen D. J. (Hoboken, NJ: John Wiley & Sons, Inc), 65–126. 10.1002/9780470939390.ch2

[B72] Trevarthen C. Delafield-Butt J. T. (2013). “Biology of shared meaning and language development: regulating the life of narratives,” in *The Infant Mind: Origins of the Social Brain*, eds Legerstee M. Haley D. Bornstein M. (New York, NY: Guildford Press), 167–199.

[B73] Trevarthen C. Delafield-Butt J. T. (2015). “The infant’s creative vitality, in projects of self-discovery and shared meaning: how they anticipate school, and make it fruitful,” in *International Handbook of Young Children’s Thinking and Understanding*, eds Robson S. Quinn S. F. (Abingdon, Oxfordshire & New York, NY: Routledge).

[B74] Trevarthen C. Delafield-Butt J. T. (2017). “Development of consciousness,” in *Cambridge Encyclopedia of Child Development*, eds Hopkins B. Geangu E. Linkenauger S. (Cambridge: Cambridge University Press), 821–835. 10.1017/9781316216491.131

[B75] Trevarthen C. Hubley P. (1978). “Secondary intersubjectivity: confidence, confiding and acts of meaning in the first year,” in *Action, Gesture and Symbol: the Emergence of Language*, ed. Lock A. (London: Academic Press), 183–229.

[B76] Trivers R. (1974). Parent-offspring conflict. *Am. Zool.* 14 249–264. 10.1093/icb/14.1.249

[B77] Wright P. (1989). “Feeding experiences in early infancy,” in *Handbook of the Psychophysiology of Human Eating*, ed. Shepherd R. (Chichester: John Wiley), 157–178.

